# Supplementary material for: Positively charged mineral surfaces promoted the accumulation of organic intermediates at the origin of metabolism
Source: PLoS Comput Biol. 2022 Aug 17;18(8):e1010377. doi: 10.1371/journal.pcbi.1010377 (PMC9423644; doi:10.1371/journal.pcbi.1010377)
Supplement: S1 Fig — The model comprises three computational domains, namely the cell, membrane, and ocean. Maxwell’s first law and species mass-balance equations are solved in the cell and membrane to ascertain the electric-potential field and concentration distributions. However, the surface potential on the outer surface of the membrane, electric-potential field, and concentration distributions in the ocean are approximated by the Gouy-Chapman theory [52, Section 5.3]. (PDF) [file pcbi.1010377.s001.pdf]

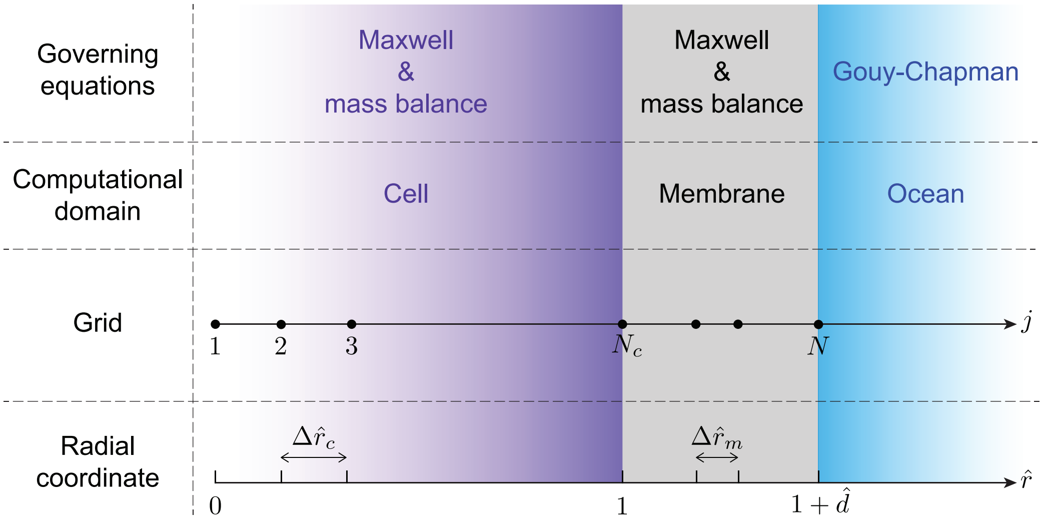

Figure S1: Computational domains in the protocell model of life's origins described in Fig 1 and the grid used to discretize the governing equations. The model comprises three computational domains, namely the cell, membrane, and ocean. Maxwell's first law and species mass-balance equations are solved in the cell and membrane to ascertain the electric-potential field and concentration distributions. However, the surface potential on the outer surface of the membrane, electric-potential field, and concentration distributions in the ocean are approximated by the Gouy-Chapman theory.
