# Supplementary material for: Positively charged mineral surfaces promoted the accumulation of organic intermediates at the origin of metabolism
Source: PLoS Comput Biol. 2022 Aug 17;18(8):e1010377. doi: 10.1371/journal.pcbi.1010377 (PMC9423644; doi:10.1371/journal.pcbi.1010377)
Supplement: S2 Fig — (A) Electric potential, (B) volume-charge density, (C) concentration of cations and anions associated with salt-I, and (D) concentration of cations and anions associated with salt-II. Here, C∞,i denotes the far-field concentration of ion i in the ocean, arising from dissociation of the respect salt in water. Shaded areas indicate the position of the membrane along the r-axis. The scale on the r-axis, where the ocean lies, is stretched by a factor 20 to better show radial profiles. (PDF) [file pcbi.1010377.s002.pdf]

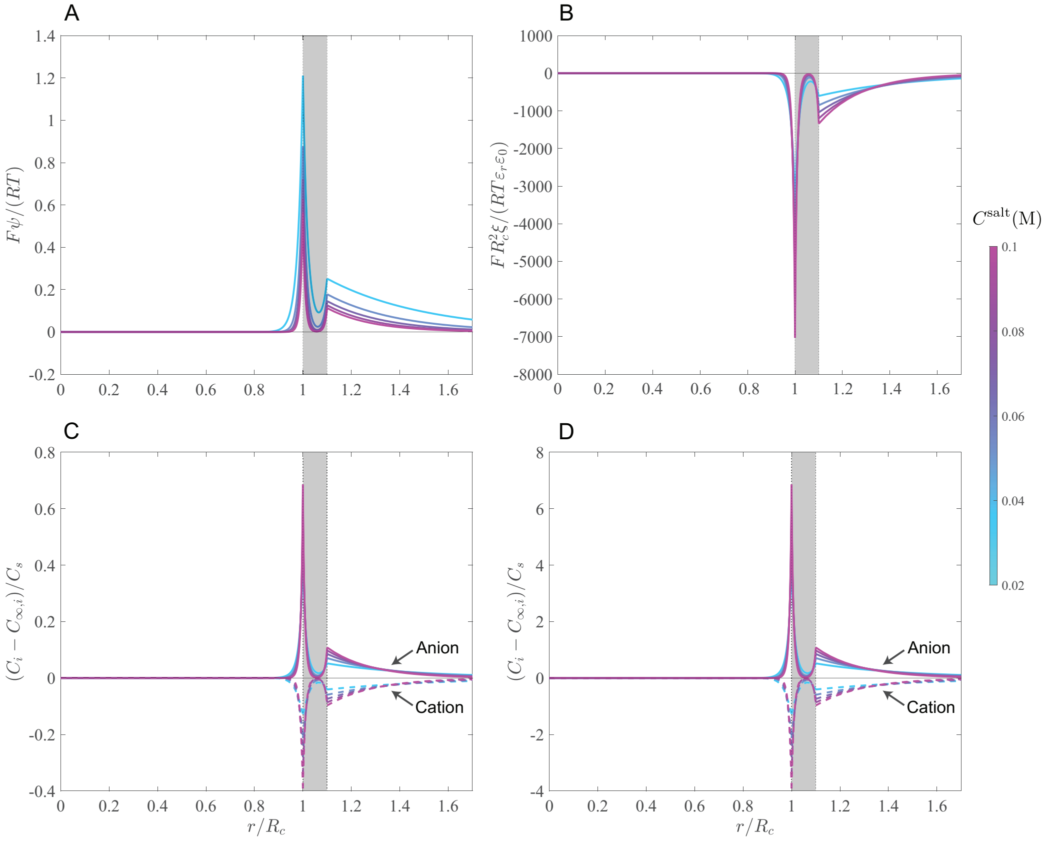

Figure S2: Steady-state solutions of species mass-balance and Maxwell's first equations at $R_c = 10^{-7}$ m, $\sigma = 0.01$ C/m^2, and $\sigma_r = 0.2$. (A) Electric potential, (B) volume-charge density, (C) concentration of cations and anions associated with salt-I, and (D) concentration of cations and anions associated with salt-II. Here, $C_{\infty,i}$ denotes the far-field concentration of ion i in the ocean, arising from dissociation of the respect salt in water. Shaded areas indicate the position of the membrane along the r-axis. The scale on the r-axis, where the ocean lies, is stretched by a factor 20 to better show radial profiles.
