# Supplementary material for: Positively charged mineral surfaces promoted the accumulation of organic intermediates at the origin of metabolism
Source: PLoS Comput Biol. 2022 Aug 17;18(8):e1010377. doi: 10.1371/journal.pcbi.1010377 (PMC9423644; doi:10.1371/journal.pcbi.1010377)
Supplement: S3 Fig — (A) Rc = 10−6 m and ϑ = 0.05, (B) Rc = 10−8 m and ϑ = 0.05, (C) Rc = 10−6 m and ϑ = 0.1, and (D) Rc = 10−8 m and ϑ = 0.1. Colorbars indicate the value of Csalt = C∞/2 that corresponds to each curve in (A)–(D). The tortuosity coefficient ϑ only affects stability without altering steady-state solutions. Solid and dashed lines represent stable and unstable steady-state solutions, respectively. (PDF) [file pcbi.1010377.s003.pdf]

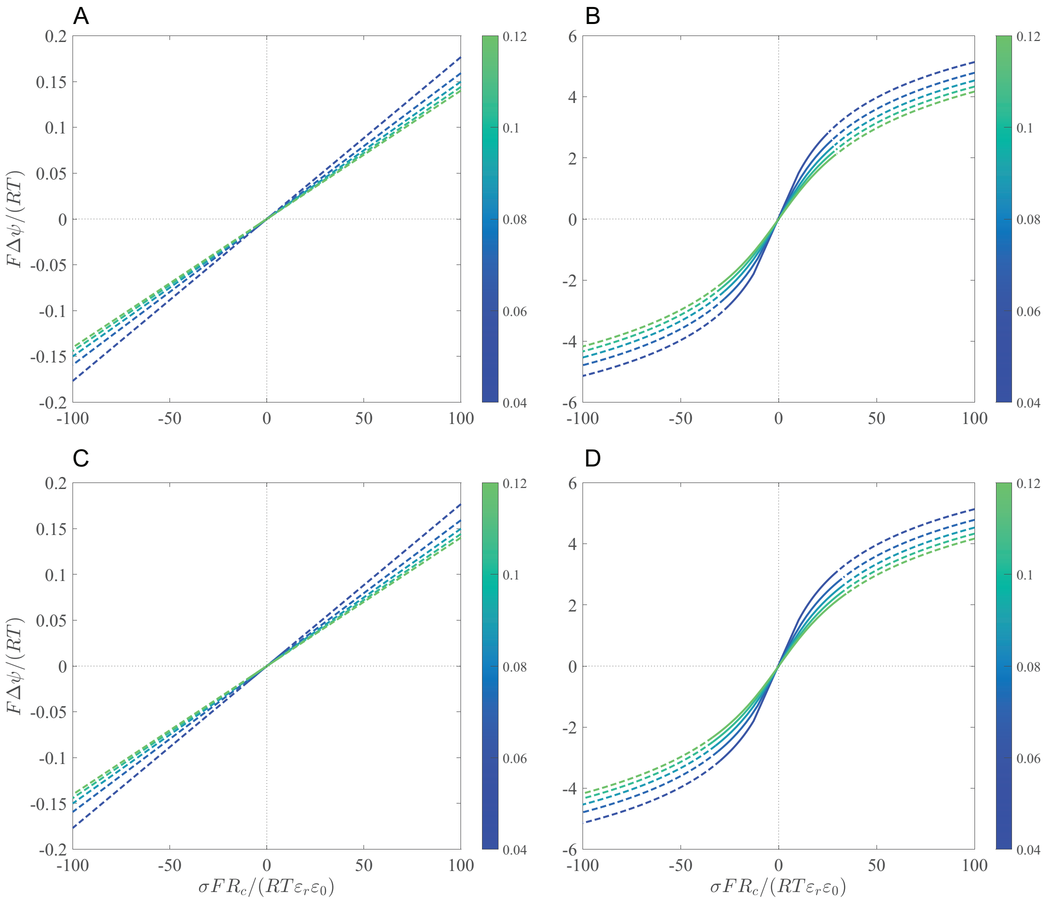

Figure S3: Stability along steady-state solution branches of $\Delta\psi$ parametrized with respect to the surface-charge density σ at $\sigma_r = 0.02$ with (A) $R_c = 10^{-6}$ m and $\vartheta = 0.05$, (B) $R_c = 10^{-8}$ m and $\vartheta = 0.05$, (C) $R_c = 10^{-6}$ m and $\vartheta = 0.1$, and (D) $R_c = 10^{-8}$ m and $\vartheta = 0.1$. Colorbars indicate the value of $C^{\text{salt}} = C_\infty/2$ that corresponds to each curve in (A)–(D). The tortuosity coefficient ϑ only affects stability without altering steady-state solutions. Solid and dashed lines represent stable and unstable steady-state solutions, respectively.
