# Supplementary material for: Positively charged mineral surfaces promoted the accumulation of organic intermediates at the origin of metabolism
Source: PLoS Comput Biol. 2022 Aug 17;18(8):e1010377. doi: 10.1371/journal.pcbi.1010377 (PMC9423644; doi:10.1371/journal.pcbi.1010377)
Supplement: S4 Fig — (A) surface-charge density, at which stability is lost. (B) Membrane potential, at which stability is lost. (PDF) [file pcbi.1010377.s004.pdf]

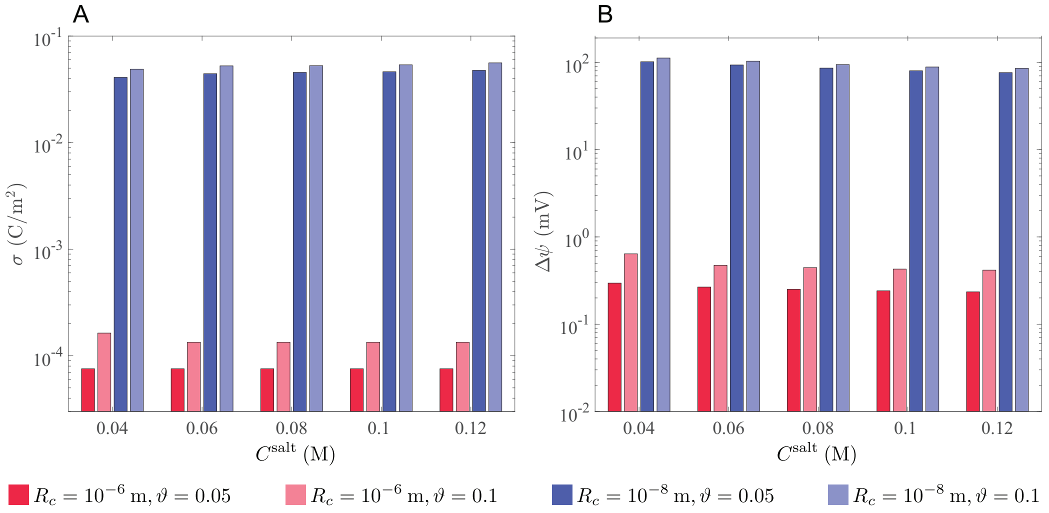

Figure S4: Stability limits along steady-state solution branches of Fig 3 in the positive orthant. (A) surface-charge density, at which stability is lost. (B) Membrane potential, at which stability is lost.
