# Supplementary material for: Positively charged mineral surfaces promoted the accumulation of organic intermediates at the origin of metabolism
Source: PLoS Comput Biol. 2022 Aug 17;18(8):e1010377. doi: 10.1371/journal.pcbi.1010377 (PMC9423644; doi:10.1371/journal.pcbi.1010377)
Supplement: S5 Fig — (A) Rc = 10−6 m and (B) Rc = 10−8 m. Each Thiele modulus ΛB corresponds to a solid-dashed curve pair, increasing along the direction indicated by the arrows. Red lines represent the nonreactive limit, where ΛB → 0. (PDF) [file pcbi.1010377.s005.pdf]

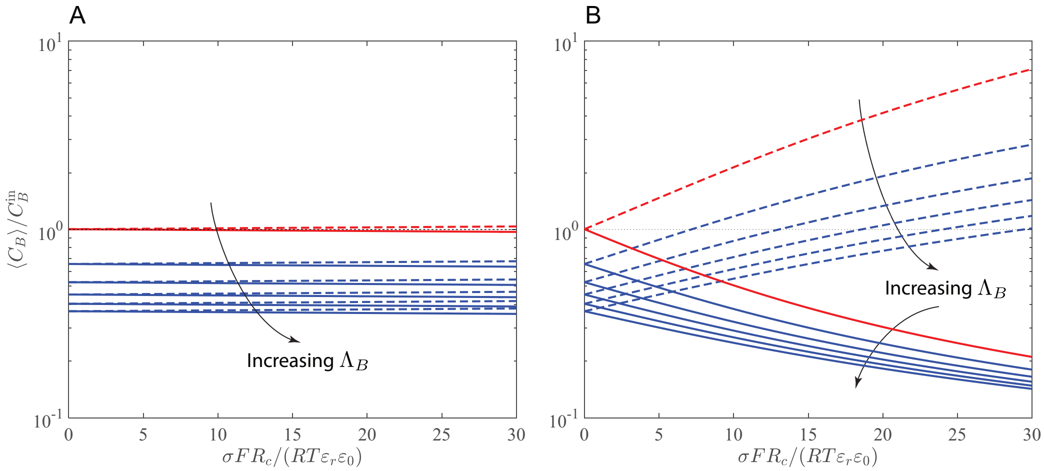

Figure S5: Average concentration of the cation (dashed lines) and anion (solid lines) arising from the dissociation of a monovalent salt inside the cell at $C^{\mathrm{salt}} = 0.1$ M, $\Lambda_B^2 = 0, 10, 20, 30, 40, 50$, and (A) $R_c = 10^{-6}$ m and (B) $R_c = 10^{-8}$ m. Each Thiele modulus Λ_B corresponds to a solid-dashed curve pair, increasing along the direction indicated by the arrows. Red lines represent the nonreactive limit, where $\Lambda_B \to 0$.
