# Supplementary material for: Positively charged mineral surfaces promoted the accumulation of organic intermediates at the origin of metabolism
Source: PLoS Comput Biol. 2022 Aug 17;18(8):e1010377. doi: 10.1371/journal.pcbi.1010377 (PMC9423644; doi:10.1371/journal.pcbi.1010377)
Supplement: S6 Fig — A pH gradient across the membrane of the protocell model shown in Fig 1 is assumed to cause a surface charge-density differential between the inner and outer surfaces of the membrane. The protocell resides near a hydrothermal vent at an interface between two fluids with pH ≈ 9 and pH ≈ 5. The fluids flow into the cell from the alkaline vent and acidic ocean. The acidic and alkaline fluids neutralize into water, such that pH ≈ 7 in the cell. Mineral-I and Mineral-II are two hypothetical minerals that exhibit different functional forms for σ(pH). For the given pH gradient between the hydrothermal vent and ocean, the surface-charge densities formed on the inner and outer surfaces of a membrane made of Mineral-I are almost identical. However, a large surface-charge density differential can be generated across a membrane made of Mineral-II, such that σin and σout are both positive, similarly to the scenario described in the left diagram of Fig 1B. (PDF) [file pcbi.1010377.s006.pdf]

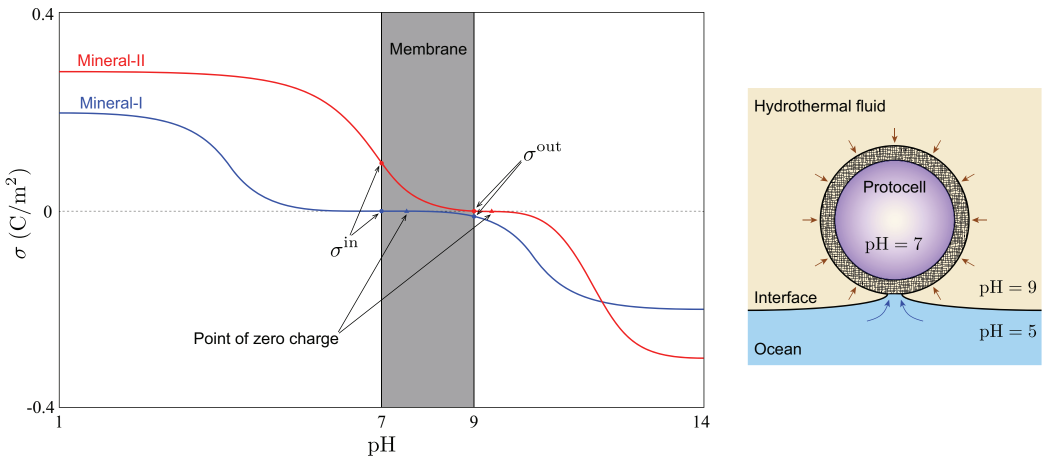

Figure S6: Typical surface-charge density of minerals measured as a function of pH using potentiometric-conductometric titration experiments. A pH gradient across the membrane of the protocell model shown in Fig 1 is assumed to cause a surface charge-density differential between the inner and outer surfaces of the membrane. The protocell resides near a hydrothermal vent at an interface between two fluids with pH $\approx$ 9 and pH $\approx$ 5. The fluids flow into the cell from the alkaline vent and acidic ocean. The acidic and alkaline fluids neutralize into water, such that pH $\approx$ 7 in the cell. Mineral-I and Mineral-II are two hypothetical minerals that exhibit different functional forms for σ(pH). For the given pH gradient between the hydrothermal vent and ocean, the surface charge densities formed on the inner and outer surfaces of a membrane made of Mineral-I are almost identical. However, a large surface charge-density differential can be generated across a membrane made of Mineral-II, such that σ^{in} and σ^{out} are both positive, similarly to the scenario described in the left diagram of Fig 1B.
