# Supplementary material for: Positively charged mineral surfaces promoted the accumulation of organic intermediates at the origin of metabolism
Source: PLoS Comput Biol. 2022 Aug 17;18(8):e1010377. doi: 10.1371/journal.pcbi.1010377 (PMC9423644; doi:10.1371/journal.pcbi.1010377)
Supplement: S1 Table — (PDF) [file pcbi.1010377.s008.pdf]

Table S1: Parameters used for all the case studies presented in the main text and supplementary information.

Parameter	Value	Parameter	Value
T	145° C	D_1^+ (salt-I)	2×10^{-9} m^2/s
$\varepsilon_r^\dagger$	50.97	D_1^- (salt-I)	2×10^{-10} m^2/s
r_{12}	0.1	D_2^+ (salt-II)	8×10^{-10} m^2/s
D_s	10^{-9} m^2/s	D_2^- (salt-II)	10^{-10} m^2/s
C_s	10^{-2} M		
$\hat{d}$	0.1		

†Estimated using the revised Helgeson-Kirkham-Flowers equation of state at 100 bar and in the temperature range 120–145° C.
