# Supplementary material for: Positively charged mineral surfaces promoted the accumulation of organic intermediates at the origin of metabolism
Source: PLoS Comput Biol. 2022 Aug 17;18(8):e1010377. doi: 10.1371/journal.pcbi.1010377 (PMC9423644; doi:10.1371/journal.pcbi.1010377)
Supplement: S2 Table — Expressions in the Scheme column are derivatives evaluated at r^j, where fj′≔f′(r^j). Note that Δr^≔Δr^c when 1 ≤ j ≤ Nc and Δr^≔Δr^m when Nc + 1 ≤ j ≤ N (see S1 Fig). (PDF) [file pcbi.1010377.s009.pdf]

Table S2: Forth-order finite-difference schemes to approximate the first derivative of a function $f(\hat{r})$ on the grid shown in S1 Fig. Expressions in the Scheme column are derivatives evaluated at $\hat{r}_j$, where $f'_j := f'(\hat{r}_j)$. Note that $\Delta\hat{r} := \Delta\hat{r}_c$ when $1 \leq j \leq N_c$ and $\Delta\hat{r} := \Delta\hat{r}_m$ when $N_c + 1 \leq j \leq N$ (see S1 Fig).

Index	Scheme
$j = 1$	$\frac{-25f_j+48f_{j+1}-36f_{j+2}+16f_{j+3}-3f_{j+4}}{12\Delta\hat{r}}$
$j = 2,\ N_c + 1$	$\frac{-3f_{j-1}-10f_j+18f_{j+1}-6f_{j+2}+f_{j+3}}{12\Delta\hat{r}}$
$\begin{cases} 3 \leq j \leq N_c - 2 \\ N_c + 2 \leq j \leq N - 2 \end{cases}$	$\frac{f_{j-2}-8f_{j-1}+8f_{j+1}-f_{j+2}}{12\Delta\hat{r}}$
$j = N_c - 1,\ N - 1$	$\frac{-f_{j-3}+6f_{j-2}-18f_{j-1}+10f_j+3f_{j+1}}{12\Delta\hat{r}}$
$j = N_c,\ N$	$\frac{3f_{j-4}-16f_{j-3}+36f_{j-2}-48f_{j-1}+25f_j}{12\Delta\hat{r}}$
