# Supplementary material for: Positively charged mineral surfaces promoted the accumulation of organic intermediates at the origin of metabolism
Source: PLoS Comput Biol. 2022 Aug 17;18(8):e1010377. doi: 10.1371/journal.pcbi.1010377 (PMC9423644; doi:10.1371/journal.pcbi.1010377)
Supplement: Attachment — Submitted filename: Response to Reviewers.docx [file pcbi.1010377.s011.docx]

**Response to Editor’s comments:**

I recommend a major revision for your manuscript, I would like however ask you not only to address the specific comments of the reviewers, but also, for revising the manuscript to make it more appealing and more accessible to the biochemists and the biologists – which after all, are the key audience you need to convince if your paper is to create impact.

We appreciate the editor’s comments. Our point-by-point responses are provided below.

Two Editorial suggestions in this regard:

In the first part of the discussion, the manuscript talks about the dispute that existed in the literature about whether protometabolic #cycles# would be chemically feasible. You quote some well-known, but 15+ year old ‘opinion -type’ papers -- but there is a significant amount of more recent, experimental, literature, that actually discovered that Fe(II) and other transition metals catalyze metabolism like reactions, that mimic glycolysis, pentose phosphate pathway and Krebs cycle. It might help your manuscript to set the scene around experimentally proven facts, rather then speculative review-type pieces by Orgel and co, that divided the filed a long time ago. Specifically, it may indeed appeal to the biologists if they are reminded that there is experimental evidence that glycolysis and the TCA -like reactions are catalyzed by metal irons.

We agree with the editors that recent experimental works weaken the basis of the argument presented by Orgel and others against the metabolism-first hypothesis. To address this comment, we have rewritten a large part of the introduction in the revised manuscript, where we discuss the experimental work that the editor is referring to (highlighted on page 2-3 of the revised manuscript). We also removed the text at the beginning of the “Discussion” section that the editor is referring to avoid confusion about what the main message of the paper is. In the revised manuscript, we begin the “Discussion” section by directly stating the main focus of the paper, which is addressing the concentration problem, by proposing a mechanism based on positive membrane potentials (highlighted on page 17 of the revised manuscript).

Btw, does it really need to be ‘cycles’ (in the metabolic network sense), or do reaction sequences suffice?

This is an interesting question. Given the important role of anabolism at the origin of life, the goal of many research groups working on the metabolism-first hypothesis is to show that metabolic pathways originated from some primitive versions of modern autotrophic pathways in modern organisms that could have spontaneously emerged on the early Earth. From the six known autotrophic pathways, five are cyclic/autocatalytic [1]. This might be one of the reasons why first metabolic pathways are believed to be cyclic. Another reason is that cyclic pathways, such as the reductive tricarboxylic acid cycle (rTCA), can be fully autocatalytic, meaning that they can operate without needing any substrate but CO_2_ and H_2_ [2-4]. Of course, these cycles also need some inorganic catalysts and reducing agents; however, these compounds are assumed to be readily available in the environment in which they originated, so they are in the food set. The rTCA cycle is a good example. To start, the cycle needs acetate [4]. However, once it is stabilized, it can operate by fixing CO_2_ alone without needing acetate, so it becomes fully autotrophic [2-3]. Autocatalytic cycles are also self-amplifying [1-3], so they have the potential to evolve into more complex chemical reaction networks.

This question can also be answered on other levels. In the first level analysis, one may study the feasibility of non-enzymatic linear and cyclic pathways that could have operated under prebiotic conditions. In the second level, one can examine their dynamic features to identify some characteristics that could have underlain their evolutionary selection. For example, one can examine how stable and robust these pathways are and whether one is more optimal than others in some sense [1]. Relationships between topological features of chemical reaction networks and their dynamical characteristics, such as robustness, have been studied in the literature, for example in [5]. Although we have not exhaustively searched the literature for such relationships, cyclic networks might exhibit more robust dynamic behaviors than linear ones under a wider range of parameters associated with their environmental conditions, which might be a reason for their evolutionary selection.

Second, what might help is the focus the manuscript not on the overly broad topic of the origin of life in general, but around the specific problem about the origins of the metabolic pathways, and the metabolic network topology. There is an argument that these processes are connected, but the manuscript might sound much more tangible if it would discuss specifically the origin of the extant metabolic network structure (which may or may not be the origin of life - its not essential to go into this debate in this specific manuscript - we know as a fact that the metabolic network evolved at some point (early on), it does not really matter for this manuscript if it was the first step).

We agree with the editor that the protocell model that we discuss in this paper and a subsequent one (to appear elsewhere) mostly concerns the origins of metabolism. However, as we stated in the manuscript, we have adopted the metabolism-first hypothesis, in which the origins of metabolism and life are the same. Nevertheless, to help the reader better understand the main focus of the paper, we have rewritten a large part of the introduction in the revised manuscript as suggested by the editor (highlighted on page 2-3 of the revised manuscript). The discussion is now mainly centered around the origins of metabolism. Moreover, we removed the first paragraph of “Discussion” where we talked about criticisms and opinions around the origins of life in general in the context of metabolism-first and genetics-first hypotheses. We also change the title of the paper accordingly.

**References**

[1] Muchowska, K.B., Varma, S.J. and Moran, J., 2020. Nonenzymatic metabolic reactions and life’s origins. Chemical Reviews, 120(15), pp.7708-7744.

[2] Wächtershäuser, G., 1990. Evolution of the first metabolic cycles. *Proceedings of the National Academy of Sciences*, *87*(1), pp.200-204.

[3] Orgel, L.E., 2008. The implausibility of metabolic cycles on the prebiotic Earth. PLoS biology, 6(1), p.e18.

[4] Russell, M.J. and Hall, A.J., 1997. The emergence of life from iron monosulphide bubbles at a submarine hydrothermal redox and pH front. *Journal of the Geological Society*, *154*(3), pp.377-402.

[5] Siami, M. and Motee, N., 2013, July. Robustness and performance analysis of cyclic interconnected dynamical networks. In *2013 Proceedings of the Conference on Control and its Applications* (pp. 137-143). Society for Industrial and Applied Mathematics.

**Response to Reviewer #1’s comments:**

We thank the reviewer for their comments. Our point-by-point response to the reviewer’s comments are provided below.

Akbari and Palsson propose that positive membrane potentials caused by the accumulation of transition metals on the surface could have elevated the concentration of organic intermediates in a protometabolism at the earliest stages of the origin of life. They propose a mathematical relationship between the membrane potential at steady-state and the surface -charge density, including radial profiles. They show a trade-off between the stability of the membrane and the sensitivity of the membrane potential to surface-charge density. It is concluded that electroneutrality would be advantageous to a protocell with a protometabolism because osmotic pressure and concentration heterogeneity would be minimized.

The general idea of this paper is similar to the idea previously proposed by Wächtershäuser in his theory of surface metabolism (ref. 6), as acknowledged by the authors. However, Wächtershäuser’s proposal was based purely on intuition, whereas the model treated here is mathematical and quantitative in nature. This puts this work on a much more solid level. In general, I found this paper well written, well-reasoned and well referenced. I could recommend it for publication.

I have a few comments that the authors might want to consider addressing, at their discretion.

-Some of the ideas regarding the “concentration problem” in protometabolism have also been expressed with regards to the polymerization of monomers. In a 1951 book cited below, Bernal suggested that concentrations of the products, which would be an absolute necessity for evolution, could be attained by adsorption on very fine clay deposits in marine and fresh water.

Book: J. D. Bernal: The Physical Basis of Life. Routledge and Kegan Paul, London 1951.

An experimental example of this proposal was later shown by Katchalsky.

Paecht-Horowitz, M.; Berger, J.; Katchalsky, A. Prebiotic Synthesis of Polypeptides by Heterogeneous Polycondensation of Amino-Acid Adenylates. Nature 1970, 228 (5272), 636-639. https://doi.org/10.1038/228636a0.

We have discussed the mechanism proposed by Bernal and cited the references that the reviewer suggested in the revision (highlighted on page 15 of the revised manuscript).

-Although Wächtershäuser is often credited for the igniting the field of protometabolism, one of the first was Granick.

Granick, S. SPECULATIONS ON THE ORIGINS AND EVOLUTION OF PHOTOSYNTHESIS. Ann. NY Acad Sci. 1957, 69, 292-308. https://doi.org/10.1111/j.1749-6632.1957.tb49665.x.

We have cited this paper in the introduction of the revised manuscript (in first paragraph of the introduction of the revised manuscript).

-On pg. 3, the authors state “metabolic reactions could not have proceeded fast enough to synthesize necessary precursors for the evolution of complex reaction networks without enzymes or genetic replication.” I don’t know on what basis this claim can be made. Very few experimentalists have worked on protometabolism, so this conclusion is difficult to reach in the absence of experimentation.

In this paper and an accompanying paper (to appear elsewhere), we adopted the metabolism-first hypothesis. As we stated in the introduction, a key assumption in this hypothesis is that enzymes did not exist when the first metabolic cycles emerged on the primitive Earth. Within this framework, it is widely accepted in the literature that complex enzymes, such as ATP synthase, electron transport chain redox carriers, and ion transporters are the products of a long evolutionary process. Thus, the operation of the earliest cells at the origin of life could not have relied on these machineries and mechanisms in any way. The main challenge of constructing a theoretical model of the origin of metabolism then is to propose a mechanism by which such primitive cells could have spontaneously arisen and operated without relying on the foregoing mechanisms and proteins.

The purpose of this paragraph in the introduction that the reviewer is referring to is to highlight the main challenges that primitive cells must have faced if the complex machinery and processes that modern cells depend on to survive are eliminated. Once these challenges have been laid out, we proceed to clarify the specific objective of the paper, which is addressing those challenges associated with the concentration problem in the last paragraph of the introduction. Although these challenges may seem extremely difficult or even impossible to overcome in a primitive cell made purely of inorganic compounds at first sight, our goal is to introduce alternative mechanisms within the restrictive framework outlined in the “Model Description” section and demonstrate their feasibility through quantitative analyses. So, the claim here is not that such alternative mechanisms do not exist or have not been previously examined in the literature. We are aware of previous works on possible prebiotic mechanisms to address some of these challenges.

The specific point raised by the reviewer here is an example of the type of challenges primitive cells must have overcome. The point we are trying to make is that because the rates of reactions catalyzed by inorganic catalysts would have been much lower than the enzymatic counterparts and the fact that inorganic membranes would have been less effective a barrier than lipid bilayers, overcoming the concentration problem would have been much more difficult in primitive cells than modern cells. To avoid the kind of confusion that the wording of this paragraph may lead to, we have rewritten a large part of the introduction in the revised manuscript (highlighted on page 2-3 of the revised manuscript).

--On pg. 3 “primitive cells lacking lipid membranes”. Perhaps the authors should define exactly what they mean by primitive cells. Lots of readers would assume this involves a lipid membrane, so precisely defining what is meant here could be useful.

We have added a paragraph in the revised manuscript to further clarify what is meant by primitive cells in this paper (highlighted on page 4-5 of the revised manuscript).

Regarding the impact of elevated metabolite concentrations on catalysis, in general a problem in catalysis is the inhibition of the catalyst by binding to the product. If the membrane potential increases metabolite concentrations too much, could product-inhibition become more problematic than the problem of low concentrations? Where might this trade-off happen?

This is an interesting point. We may examine the inhibition of catalytic reactions at three levels: (i) Thermodynamics, (ii) catalyst saturation associated with the relative value of product concentrations and the respective Michaelis constants K_m_, as discussed for example in [1], and (iii) binding of catalyst with an inhibitor. The main focus of this paper is on addressing the concentration problem by studying the distribution of nonreactive species (e.g. inorganic ions like K^+^, Na^+^, Cl^-^,…) subject to an electric field. We argued that because of the low concentrations of the organic products of early metabolic cycles compared to the concentration of nonreactive species, we could decouple the mass-balance equations for reactive and nonreactive species and study them separately. So, in this paper, we did not explicitly deal with reaction-diffusion systems that arise if catalytic reaction rates of early chemical reaction networks are accounted for.

However, in an accompanying paper (to appear elsewhere), we examined the steady-state solutions of mass-balance equations for reactive species for a core metabolic network model of early metabolic cycles. For catalytic reaction rates, we accounted for the effect of product inhibition when the concentrations of reactants/products reached their thermodynamic equilibrium levels. We also accounted for catalyst substrate/product saturation by considering a simplified version of the fractional saturation term in the rate laws discussed in [1]. We did not treat any of the reactants in the network as an inhibitor for any of the reactions in the network.

An important example relevant to the point the reviewer has raised here was the concentration of CO_2_ in our model. Since the metabolic cycle that we considered involved consecutive steps of carboxylation and decarboxylation, high or low concentrations of CO_2_ in the protocell inhibited the flux through the cycle because of thermodynamic equilibrium and product saturation. For the particular set of turnover numbers and Michaelis constants we considered, we found that $[{CO}_{2}]\approx1mM$ maximized the flux through the cycle. Since the system of mass-balance equations for early metabolic networks are generally nonlinear, the inhibitory effects of different reactants in the system could influence different parts of the network to a varying degree and are nontrivial to predict. Therefore, to determine where the trade-off between substrate promotion and product inhibition occurs, one should analyze the specific reaction network of interest by performing concrete computations and solving the mass-balance equations. In general, intermediate values of the concentration of reactants that have counteracting effects on different steps of the network, such as CO_2_ in our metabolic cycle, lead to an optimal flux state.

-Pg. 8, “the total salt concentration in the ocean”. Why does this have to be in the ocean? Isn’t that too specific a location for this work? Same goes for pg. 15.

The total salt concentration $C^{salt}$ is associated with the environment, in which primitive cells reside. The early ocean is a possible environment that we considered in this paper, but it is not a strictly necessary assumption. The term ‘ocean’ in this paper refers to a computational domain in our idealized model as shown in Fig. S1. Accordingly, the parameters associated with the ocean in our model, such as $C^{salt}$, should be regarded as characterizing the ambient of primitive cells. We have clarified this point in the revised manuscript (highlighted on page 3 of the revised manuscript).

-Wächtershäuser brought up the idea that polyanionic compounds would bind more favorably to a positively charged surface, which makes sense. How does the number of negative charges quantitatively influence this model?

The number of negative charges affect the transport rate of ions through the membrane, and thus the steady-state concentrations. In our model, since there are no specialized ion transporters, the only mechanism of membrane transport is unselective passive diffusion subject to an electric field. The membrane potential affects the transport rate of all the ions simultaneously, but to a varying degree depending on their charge.

**References**

[1] Noor, E., Flamholz, A., Liebermeister, W., Bar-Even, A. and Milo, R., 2013. A note on the kinetics of enzyme action: a decomposition that highlights thermodynamic effects. *FEBS letters*, *587*(17), pp.2772-2777.

**Response to Reviewer #2’s comments:**

We thank the reviewer for their comments. Our point-by-point response to the reviewer’s comments are provided below.

In their manuscript “Positively charged mineral surfaces promoted the accumulation of organic intermediates at the origin of life” Akbari and Palsson study the effects of different properties of potential protocell surfaces/membrane constitutions on the capacity to concentrate organic substances.

Unfortunately, I have to say that after studying the manuscript I was left more confused than I was before reading it. It was incredibly difficult to find a “red line”, because apart from some occasional bright spots, the entire manuscript has no intuitively structured.

The erratic narrative style and the mixing of introductory parts and results contribute in particular to this. Assuming that because of my research background, I would be one of the readers who should be particularly interested in the topic, it is to be assumed that the paper in its current form will hardly be read and even less understood. I cannot recommend the manuscript for publication. The Authors are strongly advised to ask their direct peers for critical and honest feedback, always with the aim of making the context understandable to a potential reader.

The main message that we are trying to convey in this paper is that a mechanism based on a positive membrane potential, which could have been induced across the iron-sulfur membranes of primitive cells by a pH gradient, could have plausibly addressed the concentration problem at the origin of life. We believe the reader can find this message in the title, abstract, introduction (last paragraph), and discussion (second paragraph). In the main body of the manuscript, we proceed to quantitatively show the feasibility of this mechanism from first principles in a protocell model.

The proposed mechanism is consistent with Wachtershauser’s theory of surface metabolism and is compatible with the conditions found in alkaline hydrothermal vents with regards to materials (iron-sulfur minerals and transition metals) and physico-chemical conditions (pH gradient). The extracellular environment of our proposed protocell model is alkaline (pH=9), while the intracellular environment is acidic/neutral (pH=5-7). We discussed in the “Surface Charge of Minerals” section of Materials and Methods how these conditions could induce a positive membrane potential across membranes made of transition metals.

In an accompanying paper (to appear elsewhere), we used the same protocell model and showed that, in fact, positive membrane potentials beyond a minimum level are necessary to ensure that a steady-state flux passes through an entire autotrophic network model of early metabolic cycles. This network is catalyzed by inorganic catalysts with turnover numbers that are several orders of magnitude smaller than their enzymatic counterparts. The reductive carboxylation steps of this metabolic cycle are driven by protoferredoxins on the inner surface of the iron-sulfur membrane in an acidic environment, where they are thermodynamically favorable. The oxidized forms of protoferredoxins are regenerated on the outer surface of the membrane using hydrogen as the reducing agent in an alkaline environment. These regeneration reactions are also thermodynamically favorable in alkaline environments. Thus, the proposed mechanism, in addition to the foregoing self-consistencies, also provides a plausible explanation for how prebiotic energy sources, such as pH gradient, could have been coupled to energy demanding carbon-fixation steps of early metabolic cycles in a simple manner without needing complex machineries, such as ATP synthase or proton pumps.

Taken together, we believe that these two papers furnish a system-level and quantitative framework to rigorously examine the origins of metabolism from first principles. Moreover, they provide tools and techniques for the development of future generations of biophysical single-cell models. Therefore, we believe that they will guide future theoretical works on the subject and will be of interest to broad research communities of biochemists, biologists, mineralogists, physicists, and those who have been broadly involved in origins-of-life studies.

Due and to this reasons and despite my honest efforts, I cannot claim to have really understood the model presented here. That’s why my further feedback relates to a few general issues:

- Especially in the introduction, unsecured hypotheses are often presented as absolute statements. This should be avoided in any case.

We believe the reviewer is referring to the second paragraph of introduction. The purpose of this paragraph is to highlight the main challenges that primitive cells must have faced if the complex machinery and processes that modern cells depend on to survive are eliminated. We believe the reader can better understand what primitive cells should have looked like and why we are proposing the protocell model that we described in this paper if these challenges are explicitly stated at the beginning of the paper.

Once these challenges have been laid out, we proceed to clarify the specific objective of the paper, which is addressing those challenges associated with the concentration problem in the last paragraph of the introduction. Although these challenges may seem extremely difficult or even impossible to overcome in a primitive cell made purely of inorganic compounds at first sight, our goal is to introduce alternative mechanisms within the restrictive framework outlined in the “Model Description” section and demonstrate their feasibility through quantitative analyses. So, the claim here is not that such alternative mechanisms do not exist or have not been previously examined in the literature. We are aware of previous works on possible prebiotic mechanisms to address some of these challenges. To avoid the kind of confusion that the wording of this paragraph may lead to, we have rewritten a large part of the introduction in the revised manuscript (highlighted on page 2-3 of the revised manuscript).

- The concept of electroneutrality is upheld in this publication, but there are clear indications that (unlike discussed by the authors) in living cells this is not a hard principle, but rather just a general rule with many exceptions.

Electroneutrality is believed to be generally maintained in modern cells [1]. In particular, it has been noted in the literature that some of the regulatory responses to stress conditions that induce major perturbations to the charge balance of the cell, such as osmotic or acid stress, are exhibited by the cell in such a way as to restore electroneutrality (for example section Adjustment of Cytoplasmic Solvent Composition of [2] or Figure 3 of [3]). One should also appreciate the difference between local and global violation of electroneutrality. Of course, electroneutrality can be violated locally in the electric double layers developed around phospholipid bilayers, near the surfaces of enzymes/DNAs/RNAs, or inside ion transporters. However, a large portion of the volume of the cytoplasmic or periplasmic fluids is expected to remain near electroneutral. One could, in principle, integrate the local charge over the entire volume of the cytoplasmic or periplasmic fluids to check whether electroneutrality is maintained globally in the cytoplasm or periplasm. Again, the total charge inside the cytoplasmic or periplasmic fluids is expected to be near zero, if not exactly zero. Electroneutrality is also an intuitive principle from a biophysical point of view because any major violation of electroneutrality in the cytoplasmic or periplasmic fluids could generate a background electric field, which could then interfere with the transport of charged metabolites through periplasm/cytoplasm due to the resulting strong Coulomb forces.

We should also emphasize that whether or not electroneutrality is a hard constraint in modern cells has little to do with the main focus of this paper. As stated earlier, the main goal of this paper is to propose prebiotically plausible mechanisms based on positive membrane potentials that are consistent with the restrictive framework outlined in section “Model Description”, which can address the concentration problem in primitive cells. Violation of electroneutrality is a by-product of our model, which we came across during our analyses. It is a direct consequence of the restriction that we imposed on our protocell model not to have any specialized ion transporters, proton pumps, or proton-dependent energy converting machinery such as ATP synthase. Because there is no active transport system, the only mechanism of membrane transport is unselective, passive diffusion subject to an electric field. As a result of this mechanism, when the membrane potential is nonzero, the steady-state concentrations of ions inside the protocell must necessarily violate the electroneutrality constraint.

Electroneutrality in such primitive cells is expected to be violated to a greater extent than it would in modern cells, if they were not to maintain an exactly electroneutral intracellular environment. Because ion transport through many of the membrane proteins in modern cells is electrogenic (e.g. ATP synthase, proton pumps, ion transporters), modern cells could use a fraction of the energy released by highly exergonic reactions, such as those of the electron transport chain, to transport ions across the membrane through these energy-demanding electrogenic transport systems against their concentration gradients to generate a nonzero membrane potential while maintaining a nearly electroneutral cytoplasm and periplasm.

We would also like to clarify that, in this part of our response to the reviewer’s comments, we restricted the notion of modern cells to prokaryotes for simplicity, so we only talked about electroneutrality in the cytoplasm and periplasm. Of course, our discussion also generalizes to eukaryotes, where electroneutrality applies to all compartments.

We believed that this discussion about the difference between primitive cells with no active/selective transport system and modern cells in their ability to maintain electroneutrality is interesting, and it could have important implications for evolutionary trajectories from primitive to modern cells. For this reason, we included the discussion about electroneutrality and transport mechanisms in the section “Electroneutrality Provides Selective Advantage by Minimizing Osmotic Pressure and Concentration Heterogeneity” and the third paragraph of “Discussion”. We also added a paragraph in the revised manuscript to further clarify some aspects of the mechanisms of ion transport in our model and what we mean by primitive cells/protocells in this paper (highlighted on page 4-5 of the revised manuscript).

- Almost every enrichment process (as well as depletion) in living cells works through the constant investment of some form of energy (photons, ATP, synport,..). Where does the assumption come from that this should not also have applied to the first living cells?

We agree with the reviewer. However, this statement does not claim that there were no prebiotic energy sources or mechanisms to drive the energy demanding steps of first metabolic cycles. As stated earlier, the purpose of the paragraph that the reviewer is referring to is to point out the challenges that primitive cells must have faced if the complex machinery and processes that modern cells depend on to survive are eliminated. With regards to the specific statement about prebiotic energy sources, in an attempt to construct a plausible model of primitive cells from modern cells within the restrictive framework outline in section “Model Description”, we would like to remind the reader of the challenges that arise if redox-pH gradient energy converting proteins (ATP synthase and electron transport chain) and ATP-dependent carboxylase proteins are eliminated.

This discussion is more relevant to the accompanying paper (to appear elsewhere), where we explicitly account for early metabolic reactions taking place in the protocell. Indeed, we believe that it would have been challenging for a prebiotic energy converting apparatus to emerge that could have allowed the coupling of energy sources and energy-demanding steps of early metabolic cycles. For example, Wächtershäuser suggested FeS and H2S as prebiotic energy sources/reducing agents that could have driven the carbon-fixation steps of early metabolic cycles [4]. He suggested that the oxidative formation of pyrite in the following reaction could have provided sufficient reducing power to drive these steps:

FeS+H2S->FeS2+H2

However, as pointed out by Russell & Hall [5], because of the crystal structure of pyrite, it would have been extremely difficult to regenerate FeS from FeS2 by a reducing agents/energy source (e.g. H2) to establish a continuous interconversion of FeS/FeS2 redox differential and energy sources and to drive the carbon-fixation steps. Therefore, Russell & Hall suggested alternative iron-sulfur redox couples, such as mackinawite/greigite or some protoferredoxins, that can conduct electrons where the valence of iron can be easily switched.

Thus, redox couples that could have driven carbon-fixation reactions at the origin of metabolism must have simultaneously satisfied several constraints. Notable, they should have had the right crystal structure; they should have provided sufficiently large redox gradients that could have been achievable in the environment in which metabolism originated; they should have catalyzed the carboxylation steps at sufficiently large turnover numbers, and they should have been specific enough to mostly catalyze the specific steps of the metabolic cycles from which the essential building blocks of life were synthesized. As a result, one may argue that redox couples that met all these constraints would have been challenging to emerge on the primitive Earth.

We have removed this paragraph and this specific statement in the revised manuscript to avoid any confusion about plausible prebiotic energy sources. A large part of the introduction in the revised manuscript has also been rewritten (highlighted on page 2-3 of the revised manuscript).

**References**

[1] Milo, R. and Phillips, R., 2015. Cell biology by the numbers. Garland Science.

[2] Wood, J.M., 1999. Osmosensing by bacteria: signals and membrane-based sensors. Microbiology and molecular biology reviews, 63(1), pp.230-262.

[3] Stull, F., Hipp, H., Stockbridge, R.B. and Bardwell, J.C., 2018. In vivo chloride concentrations surge to proteotoxic levels during acid stress. *Nature chemical biology*, *14*(11), pp.1051-1058.

[4] Wächtershäuser, G., 1990. Evolution of the first metabolic cycles. Proceedings of the National Academy of Sciences, 87(1), pp.200-204.

[5] Russell, M.J. and Hall, A.J., 1997. The emergence of life from iron monosulphide bubbles at a submarine hydrothermal redox and pH front. Journal of the Geological Society, 154(3), pp.377-402.
